# Autoimmune disease-associated lymphomas: research progress and review

**DOI:** 10.3389/fimmu.2025.1719391

**Published:** 2026-01-06

**Authors:** Chengqian Chen, Wei Guo, Yangzhi Zhao, Xingtong Wang, Jia Li, Ying Zhang, Zhaoxia Li, Haotian Wang, Ou Bai

**Affiliations:** Department of Hematology, The First Hospital of Jilin University, Changchun, China

**Keywords:** autoimmune diseases, lymphoma, pathogenesis, risk factors, treatment

## Abstract

Autoimmune diseases (ADs) are strongly associated with a significantly increased risk of lymphoma, with the standardised incidence ratio (SIR) markedly elevated in certain conditions, most notably in Sjögren’s disease (SjD) where an SIR as high as 18.8 has been reported. The risk is particularly prominent for diffuse large B-cell lymphoma (DLBCL) and mucosa-associated lymphoid tissue (MALT) lymphoma. This review systematically elucidates the epidemiological features, pathological mechanisms, risk factors, and therapeutic strategies of ADs-associated lymphomas. Epidemiological studies have confirmed strong associations between ADs such as rheumatoid arthritis (RA), systemic lupus erythematosus (SLE), and SjD with specific lymphoma subtypes, and these associations appear to be bidirectional. Core pathogenic mechanisms involve malignant transformation driven by the immune–inflammatory continuum: chronic antigenic stimulation and the inflammatory microenvironment result in regulatory cell (Treg/Breg) dysfunction, tertiary lymphoid structure (TLS) formation, and clonal evolution. Specific autoantibodies directly contribute to oncogenesis by interfering with intracellular signalling pathways, mimicking antigenic stimulation, and forming immune complexes, while infectious agents such as Epstein–Barr virus synergistically promote malignant transformation within immunosuppressive microenvironments. Risk factors encompass intrinsic disease features, treatment-related risks and gene–environment interactions. Clinical management must balance the dual imperatives of “controlling inflammation” and “minimising treatment-related risks”. Targeted therapies, such as rituximab and BTK inhibitors, as well as haematopoietic stem cell transplantation (HSCT), have offered hope, but prognosis remains profoundly influenced by baseline immune status. Future research should focus on risk stratification guided by multi-omics, the application of novel immunotherapies in the autoimmune setting, and the optimisation of multidisciplinary care models.

## 1 Introduction

Lymphomas are malignant tumours of the immune system arising from lymph nodes and extranodal lymphoid tissues, and their occurrence is closely linked to the malignant transformation of immune cells during lymphocyte proliferation and differentiation within immune responses (1). In recent years, the incidence of lymphoma has been steadily increasing, and immune dysregulation has been recognised as a pivotal factor in its pathogenesis. ADs are now widely acknowledged as important triggers for lymphomagenesis. According to the literature, patients with ADs have a 2- to 37-fold higher risk of developing lymphoma (2). Since Mellors first reported the association between autoimmune diseases and lymphoma in 1966 (3), research on the underlying mechanisms and relevant risk factors has steadily progressed. Numerous epidemiological studies have demonstrated a significantly increased risk of specific lymphoma subtypes among patients with ADs (4–10), particularly DLBCL (4–6). This phenomenon reflects not only shared pathophysiological mechanisms but also poses challenges for clinical diagnosis, therapeutic strategies, and prognostic assessment. Large-scale studies and Mendelian randomisation analyses have confirmed a causal relationship between ADs and non-Hodgkin lymphoma (NHL) (11–13). For example, systematic reviews and meta-analyses have revealed a significantly higher incidence of primary central nervous system lymphoma (PCNSL) in patients with ADs compared to the general population (5).

Multiple mechanisms have been proposed to explain the association between ADs and lymphomas such as NHL. On the one hand, impaired apoptosis due to Fas gene mutations, aberrant NF-κB pathway activation, dysregulated BAFF expression, and persistent overexpression of cytokines (such as IL-6, IL-10, TNF-α) may jointly contribute to lymphomagenesis (14–17). On the other hand, genome-wide studies have identified that specific HLA alleles (within the MHC region), which are associated with autoimmune diseases, also confer susceptibility to NHL, suggesting shared genetic backgrounds may partly explain their co-occurrence. However, the precise pathways mediating this relationship require further elucidation (18, 19). In-depth investigations suggest that abnormal immune activation and dysregulation of regulatory cell function represent the core mechanisms linking ADs and lymphomas (20). Chronic inflammation and persistent antigenic stimulation may drive malignant B-cell transformation, while the immunosuppressive microenvironment associated with ADs further promotes lymphoma progression (13, 20). These mechanisms not only increase the risk of lymphoma development but may also influence tumour biology and therapeutic responses.

The coexistence of ADs significantly complicates clinical management and prognosis in patients with lymphoma (2, 4, 21). For instance, DLBCL patients with concomitant ADs often face more complex treatment decisions, particularly regarding the synergistic or antagonistic effects of immunomodulatory drugs and chemotherapy (22, 23). Moreover, ADs may alter the tumour microenvironment or systemic immune state, leading to differences in survival and treatment resistance (20). It is noteworthy that, apart from immune mechanisms, environmental exposures (e.g. lifestyle factors), genetic susceptibility, and age may also act as confounding factors in this association (18, 24–27). With the rapid advancement of immunotherapies, the safety and efficacy of novel modalities such as CAR-T cell therapy in the context of ADs have become pressing questions.

This review will systematically explore the epidemiological features, pathophysiological mechanisms, diagnostic approaches, and therapeutic strategies for ADs-associated lymphomas, with a particular focus on the interplay among risk factors, molecular mechanisms, and clinical practice. By integrating existing evidence—including large-scale data analyses and clinical meta-analyses—we aim to provide insights for optimising patient management and to highlight future research directions, such as the development of novel therapies targeting the immune microenvironment.

## 2 Epidemiology of autoimmune disease–associated lymphomas

The overall incidence of ADs–associated lymphomas has not been precisely reported to date; however, the association between ADs and lymphomas has been consistently confirmed by multiple studies. Patients with RA, SLE, and SjD show a significantly higher risk of lymphoma compared to the general population (9, 28–30).

Reports indicate that the risk of malignant lymphoma in RA patients is approximately twice that of the general population (31). A large UK cohort study (n = 3,771) found that RA patients had a significantly elevated incidence of cancer, with an overall risk increase of about 28% (32). The risk was particularly elevated for lung cancer, HL, and NHL. Among lymphoma subtypes, HL and NHL showed the most pronounced increases, with SIRs of 12.8 and 3.12, respectively (32). In contrast, a large Japanese cohort study (n = 66,953) found no overall increase in malignancy incidence among RA patients, but a significantly higher incidence of lymphoma compared with the general population (SIR = 3.43). Similarly, a Swedish nationwide cohort (n = 16,392) reported that RA patients had a 1.5-fold higher risk of lymphoma (aHR 1.56–1.65). Notably, treatment with biologics, including TNF inhibitors (TNFi), did not further increase risk; long-term use was even associated with reduced risk (aHR 0.67, ≥5 years of therapy). The excess risk was predominantly observed for DLBCL (aHR 2.14) and HL (aHR 3.39), while CLL risk was reduced (aHR 0.64) (33).

SLE patients are reported to have a 4- to 7-fold higher risk of lymphoma than the general population (34). A recent systematic review and meta-analysis including 24 studies showed that the standardised incidence ratios for NHL and HL in SLE patients were 4.93 and 2.60, respectively, confirming a markedly elevated risk of both entities (35). Additionally, a population-based study in Taiwan suggests a bidirectional association between SLE and NHL: the SIR for NHL occurrence in SLE patients was 4.2; the SIR for SLE occurrence in NHL patients was 2.0. The highest risk for both conditions occurred within one year of diagnosis (SIR for NHL occurrence within one year in SLE patients = 10.3; and NHL patients had an SIR of 5.7 for developing SLE within one year) (36). However, this observation warrants cautious interpretation, as the underlying mechanisms remain unclear and contradict clinical observations of reduced autoimmune disease activity in some patients following chemotherapy. This elevated early-stage risk may be associated with increased diagnostic vigilance, dramatic shifts in immune status, or other confounding factors. Its precise causal relationship and prevalence require further research to confirm.

SjD is characterised by striking cancer susceptibility, with approximately one-third of malignancies being B-cell lymphomas (37). Among ADs, SjD confers the most pronounced risk of lymphoma (38, 39). Multiple studies consistently report markedly elevated NHL risk in SjD patients. Cohort studies have documented SIRs as high as 18.8 (40), while a meta-analysis yielded a pooled relative risk (RR) of 13.76 (95% CI: 8.53–18.99) (30). In a large retrospective Chinese study of 4,880 NHL patients, SjD accounted for the highest proportion of concurrent ADs (22.1%), significantly exceeding other ADs (41), further corroborating the association. Recent research also revealed a bidirectional link between SjD and NHL: not only are SjD patients predisposed to NHL, but NHL patients also carry an elevated risk of subsequent SjD, with both directions of risk peaking within the first year after diagnosis (42). However, this epidemiological finding warrants cautious interpretation. The risk peak observed during the initial diagnostic phase suggests potential monitoring bias, where the process of diagnosing lymphoma may have increased the detection rate of underlying SjD. Furthermore, this phenomenon contradicts the typical course of remission seen in some autoimmune diseases following immunosuppressive chemotherapy. The underlying biological mechanisms and the true direction of causality remain to be elucidated through prospective studies.

In summary, the epidemiological pattern of AD-associated lymphoma exhibits clear regularities. First, lymphoma risk exhibits significant stratification across different ADs, with SjD carrying the highest risk (SIR up to 18.8), followed by SLE and RA. Second, the association demonstrates clear subtype specificity, such as the strongest link between SjD and MALT lymphoma, and between SLE and RA with DLBCL (34, 43, 44). Furthermore, risk exhibits time dependency, with significantly elevated lymphoma incidence during the early diagnostic phase of ADs. This may involve the combined effects of diagnostic vigilance, dramatic shifts in immune status, and other factors. These epidemiological characteristics provide crucial evidence for identifying high-risk populations, elucidating potential pathogenesis, and developing personalised monitoring strategies.

## 3 Associations between autoimmune diseases and lymphoma subtypes

The associations between ADs and specific lymphoma subtypes show marked heterogeneity, likely determined by the underlying immunopathological mechanisms of each ADs. Notably, although DLBCL is the most common lymphoma subtype in the general population, its SIR remains significantly higher than in the general population across multiple autoimmune diseases, further confirming the specificity of this association. For example, in RA patients, the DLBCL SIR reaches 1.79 (45); while SLE patients exhibit a higher risk of developing DLBCL compared to healthy individuals (46). These data indicate that the elevated incidence of DLBCL in AD patients is not solely attributable to its baseline prevalence in the general population, but rather reflects the significant driving role of AD-associated immune dysregulation on this specific subtype. In B cell–mediated autoimmune diseases, RA patients most frequently develop DLBCL (47, 48), and those with concurrent DLBCL often experience poorer survival (49), suggesting a strong link between RA and DLBCL. However, some studies, such as the prospective work by Geffen et al., found that the incidence of marginal zone lymphoma (MZL) (7.6%) slightly exceeded that of DLBCL (7.2%) among RA patients (2). Nevertheless, the prevalence of DLBCL remains significantly higher than in the general population (6), possibly reflecting differences in study design or populations.

In SLE patients, the incidence of lymphoma is also markedly higher, with DLBCL accounting for 50–65% of cases and representing the predominant subtype (50, 51). Within DLBCL, the germinal centre B-cell–like (GCB) subtype is most common, generally associated with a relatively favourable prognosis (46, 52). SLE has also been linked to HL, though less prominently than to DLBCL (53).

In SjD patients, the risk of NHL is substantially elevated, particularly for MALT lymphoma, with parotid gland MALT lymphoma being most strongly associated (38). Epidemiological data indicate that SjD increases the overall risk of NHL nearly sevenfold, with especially high rates of parotid MZL (54). Other B cell–mediated ADs, including autoimmune haemolytic anaemia, Hashimoto’s thyroiditis, and myasthenia gravis, are also generally associated with increased risks of DLBCL and MZL (55).

In contrast, T cell–mediated autoimmune diseases exhibit distinct association patterns, showing a stronger link with peripheral T-cell lymphoma (PTCL) (55). Specifically, patients with dermatomyositis, psoriasis, and coeliac disease have significantly elevated risks of T-cell lymphomas (55). Notably, not all ADs confer increased lymphoma risk. For instance, inflammatory bowel disease, type 1 diabetes, sarcoidosis, pernicious anaemia, and multiple sclerosis have not been definitively linked to NHL or specific lymphoma subtypes (38).

This heterogeneity suggests distinct pathogenic pathways. Evidence indicates that the associations between ADs and NHL display marked subtype specificity, rather than a universal pattern. These NHL subtypes often arise in later stages of lymphocyte differentiation, following antigen exposure, underscoring the pivotal role of antigen-driven mechanisms in ADs-associated lymphomagenesis (54).

Demographic characteristics also modulate risk. While ADs predominantly affect females, men are more susceptible to NHL (56, 57). In addition to sex, the age of ADs diagnosis strongly predicts disease severity and complications such as lymphoma. Older patients have higher lymphoma risks, but early-onset ADs also carry increased NHL susceptibility (26).

Discrepancies among studies may arise from differences in study populations, treatment regimens, or diagnostic criteria. For example, some studies suggest that the association between RA and NHL is confined to patients treated with immunosuppressants (31, 38, 58), or that RA is not linked to specific subtypes such as mantle cell lymphoma (59). Overall, the strength of ADs–lymphoma associations varies by disease type, with the most robust evidence supporting links between SjD and MALT lymphoma, and between SLE/RA and DLBCL. T cell–mediated diseases are more strongly associated with PTCL. These findings not only inform clinical surveillance strategies for high-risk patients but also provide critical insights into shared pathogenic mechanisms.

## 4 Mechanisms and risk factors underlying lymphomagenesis in autoimmune diseases

**Figure 1:**
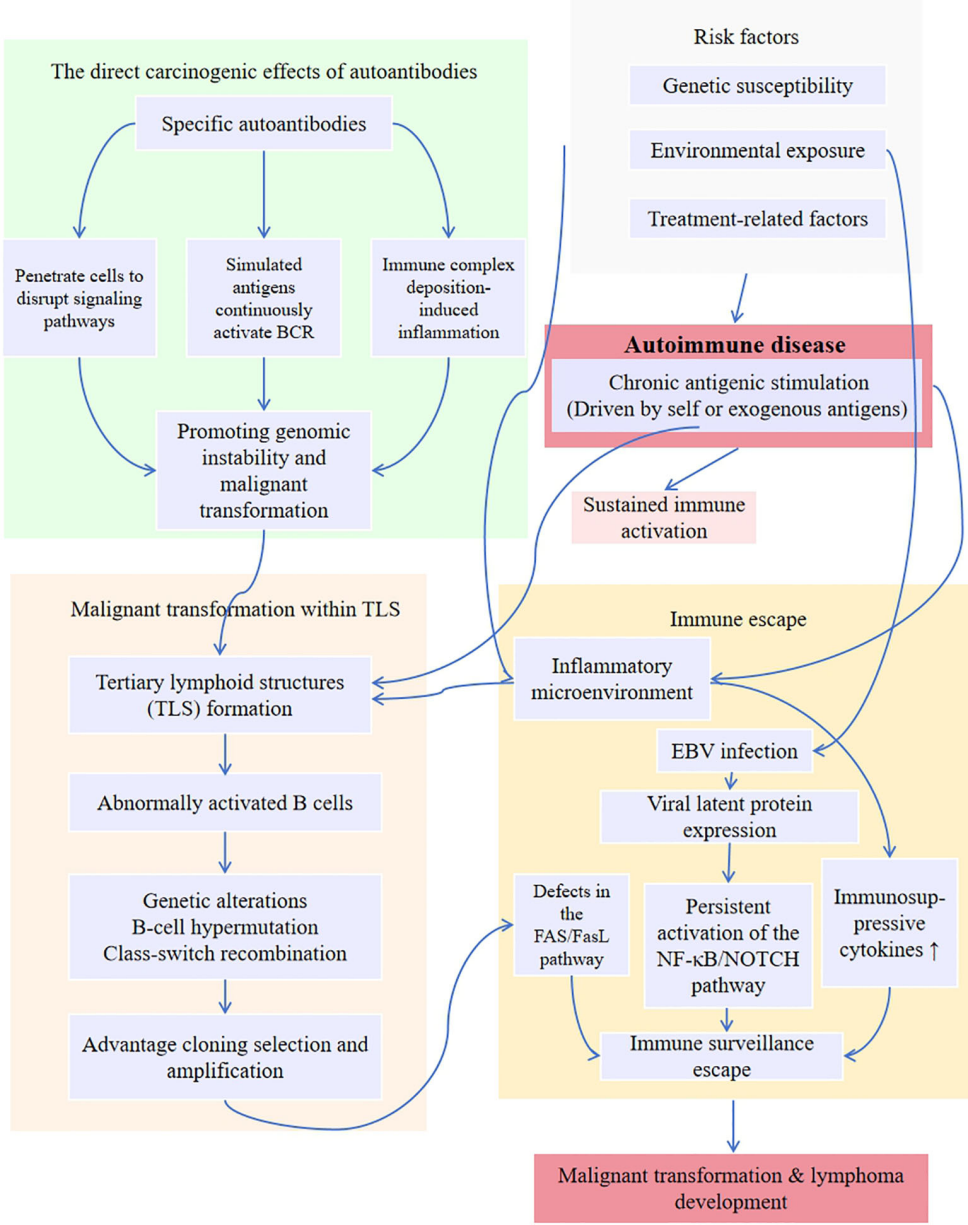
Pathogenesis of autoimmune disease-associated lymphoma.

**Figure 1** outlines the network of mechanisms underlying autoimmune-related lymphoma pathogenesis.

### 4.1 Immune-inflammatory continuum driving malignant transformation

The mechanisms by which ADs increase lymphoma risk remain incompletely understood, but among the numerous risk factors, disease severity and the degree of inflammation in ADs appear to be the most significant. Chronic inflammation and persistent antigenic stimulation during the pathophysiological processes of ADs may induce B- or T-lymphocyte proliferation, or even clonal expansion, thereby facilitating the accumulation of genetic events and increasing the risk of lymphoma (54, 60, 61). Studies on the relationship between antigens and lymphoma development suggest that lymphomagenesis is functionally driven and dynamic, rather than a simple random process. This indicates that chronic antigenic stimulation may lead to malignant transformation, with local inflammation and antigen-driven signalling promoting lymphoma progression (61, 62).

#### 4.1.1 Immune microenvironment dysregulation and clonal evolution

In ADs, the core mechanism by which chronic antigenic stimulation and the inflammatory microenvironment jointly drive lymphocyte malignant transformation lies in the abnormal clonal proliferation and evolution triggered by the imbalance of the immune microenvironment. As a chronic inflammatory disease, ADs involve prolonged hyperactivation of inflammatory mediators *in vivo*. The continuous release of inflammatory factors (such as IL-6 and TNF-α) and dysregulated immune systems can directly damage lymphocyte DNA, leading to the inactivation of tumour suppressor genes (63). Simultaneously, paracrine signalling stimulates abnormal cell proliferation, significantly increasing the risk of malignant tumours in AD patients. Furthermore, the key pro-inflammatory factor cyclooxygenase (COX)-2 and its downstream products prostaglandin E2 (PGE2), not only accelerate tumour cell proliferation, angiogenesis, and invasive metastasis but also shape a tumour-suppressive microenvironment by inhibiting effector T cell function and recruiting immunosuppressive cells (such as Tregs), playing a dominant role in tumour immune escape (64, 65).

The initiation of this malignant transformation process relies on persistent stimulation of BCR/TCR by self-antigens (e.g., salivary gland epithelial antigens in SjD) or pathogen antigens (e.g., EBV-encoded LMP1): On one hand, this stimulation directly induces constitutive activation of pro-survival signalling pathways (NF-κB, PI3K-AKT) while simultaneously upregulating the expression of anti-apoptotic proteins (e.g., BCL-XL), conferring lymphocytes with an abnormal survival capacity resistant to apoptosis (66–72). On the other hand, antigen stimulation also activates the NOTCH signalling pathway—a core pathway regulating lymphocyte development, differentiation, and immune homeostasis—further driving malignant transformation (73). As a key pathway governing immune cell fate and inflammatory responses, NOTCH frequently exhibits abnormal activation in the chronic inflammatory microenvironment of ADs: co-expression of NOTCH1, NOTCH3, and their ligands DLL1 and Jag1 in RA inflammatory synovium. Activated NOTCH1^IC^ not only promotes Th1/Th17 proliferation but also enhances IL-6 secretion by cross-linking NF-κB (17, 73). In SLE patients, M2b macrophages highly express NOTCH1^IC^, which activates the ALD-DNA pathway and promotes NF-κB nuclear translocation (17, 73); SjD salivary gland transitional type II B cells highly express NOTCH2, and their marginal zone B cell phenotype is closely associated with MALT lymphoma development (17, 73); Abnormal NOTCH activity in celiac disease intestinal T cells contributes to mucosal damage and also poses a latent risk for T-cell lymphoma malignancy (17, 73).

With persistent inflammation, the microenvironment undergoes remodelling: excessive secretion of pro-inflammatory cytokines (IL-6, TNF-α) and B-cell activating factors (BAFF/APRIL) not only maintains lymphocyte activation but also induces the formation of ectopic lymphoid tissues, known as TLSs (74–76). These TLS lack normal regulatory mechanisms yet possess functional germinal centre-like microenvironments, where follicular helper T (Tfh) cells drive somatic hypermutation (SHM) in B cells via IL-21. Continuous antigenic pressure consequently selects clones with survival advantages (77). For example, oligoclonal B-cell expansion can be detected in the salivary gland TLS of SjD patients, some of which eventually evolve into MALT lymphoma (78). In RA, TLS in synovial tissue provide an IL-23-driven microenvironment that significantly enhances clonal B-cell expansion, thereby increasing the risk of ectopic lymphoid neoplasms (ELNs) (79, 80).

Aberrant immune regulatory cell function further accelerates clonal evolution. In ADs, the suppressive capacity of regulatory T cells (Tregs) is diminished, and they may even shift toward a pro-inflammatory phenotype (e.g., secreting IFN-γ or IL-17), leading to loss of control over autoreactive B cells (81). Meanwhile, B regulatory cells (Bregs) decrease in number or function, whereas other B-cell subsets (such as activated B cells or plasmacytoid dendritic cells) secrete excessive BAFF/APRIL, further supporting autoreactive B-cell survival and antibody production (82, 83). Such a dysregulated microenvironment increases genomic instability, as AD-associated chronic antigenic stimulation drives SHM and class-switch recombination (CSR), which may cause off-target DNA damage (84). Notably, although inflammation in some ADs (e.g., RA) initially localises to specific tissues (e.g., joints), abnormal immune cell crosstalk via BAFF/APRIL signalling facilitates systemic dissemination of activated B cells, enabling lymphoma development at non-inflammatory sites such as lymph nodes or spleen (85, 86). This highlights that AD-related lymphomagenesis depends not only on local microenvironmental disruption but also on systemic immune network dysregulation.

#### 4.1.2 Immune evasion and malignant transformation

On the basis of clonal evolution, immune evasion mechanisms further drive malignant transformation. Defects in the FAS/FasL pathway represent a key mechanism (87). Under normal circumstances, FAS-mediated apoptosis eliminates excessively activated B cells and maintains immune homeostasis (88). However, in AD patients, FAS gene mutations or epigenetic abnormalities impair apoptotic clearance, allowing autoreactive B cells to persist (89). This phenomenon has been confirmed in SLE and ADs-associated lymphoma, where peripheral blood shows a significantly increased proportion of B cells with aberrant FAS expression (90). EBV infection further promotes malignant transformation within immunosuppressive microenvironments (91, 92). Overproduction of immunosuppressive cytokines such as IL-10 inhibits T-cell function, enabling EBV-infected B cells to escape immune surveillance (93). These cells, by expressing latent membrane proteins (e.g., LMP1), continuously activate the NF-κB pathway and accumulate additional oncogenic mutations, eventually leading to specific lymphoma subtypes (69, 94, 95).

The “nonlinear response model” proposed by Marc Schmidt-Supprian’s group at the Technical University of Munich provides an important theoretical framework for this process (96). According to this model, moderate B-cell activation primarily induces autoimmune pathology, whereas strong B-cell activation, if cytotoxic T lymphocyte (CTL) function remains intact, is efficiently cleared. Only when CTL dysfunction coexists with strong B-cell activation can such cells escape immune surveillance and ultimately transform into lymphoma (96). This model effectively explains a clinical paradox: certain ADs patients with high autoantibody titres (e.g., SLE) have relatively low lymphoma risk, while those receiving long-term immunosuppressive therapy (causing T-cell dysfunction) demonstrate significantly elevated risk.

In summary, ADs-associated lymphomagenesis is a multistep, dynamic evolutionary process encompassing chronic antigenic stimulation → clonal expansion → genomic instability → immune evasion → malignant transformation. Abnormal TLS microenvironments not only support clonal proliferation but also facilitate immune escape, while systemic immune dysregulation further promotes dissemination of malignant B cells. This mechanistic framework provides important insights into lymphoma risk in AD patients and suggests potential clinical intervention strategies, such as targeting BAFF or NF-κB pathways. It should be noted that the strong causal link between “chronic inflammation - clonal proliferation - malignant transformation” has been fully validated only in marginal zone lymphomas (such as MALT lymphoma). In other subtypes like DLBCL and PTCL, although correlations between inflammation and incidence have been observed, the specific molecular regulatory pathways (e.g., whether TLS formation is involved) remain incompletely elucidated. This limitation requires resolution through subsequent research.

### 4.2 Direct oncogenic roles of autoantibodies

Traditionally, autoantibodies have been regarded merely as serological markers of ADs. However, recent studies have revealed that specific autoantibodies can directly participate in oncogenic processes through multiple mechanisms, thereby serving as a crucial bridge linking autoimmunity and malignancy. These antibodies not only reflect the dysregulated state of the immune system but also actively create tumour-promoting microenvironments by disrupting key signalling pathways, mimicking antigenic stimulation, and forming immune complexes. A deeper understanding of the direct oncogenic effects of autoantibodies is of great importance for assessing lymphoma risk in ADs patients and developing early intervention strategies.

#### 4.2.1 Molecular pathway abnormalities and direct actions of autoantibodies

Autoantibodies can directly interfere with normal cellular functions via multiple mechanisms, thereby facilitating malignant transformation.

Intracellular mechanisms. Certain antibodies targeting intracellular antigens can penetrate living cells through membrane translocation or Fc receptor-mediated internalisation, subsequently disrupting critical physiological processes (97). For example, anti-RNA polymerase III antibodies, strongly associated with lymphoma in systemic sclerosis (SSc), specifically bind and inhibit the catalytic activity of RNA polymerase III, leading to dysregulation of microRNAs and other small RNAs. This results in genome-wide disturbances in protein synthesis and enhanced genomic instability (98, 99). Similarly, in dermatomyositis, anti-transcription intermediary factor 1-γ (TIF1-γ) antibodies bind TIF1-γ protein and interfere with its interaction with Smad complexes, thereby aberrantly modulating transcriptional activity of the TGF-β signalling pathway. This dysregulation impairs normal cellular growth control, differentiation, and apoptosis, thus creating favourable conditions for tumourigenesis (100, 101).

Cell-surface mechanisms. Certain autoantibodies that specifically bind to the surface of lymphocytes can act as mitogenic signals, persistently activating BCR-associated signalling cascades (102). Specifically, these autoantibodies function by mimicking natural antigens, resulting in sustained BCR pathway activation that mirrors chronic antigenic stimulation and ultimately drives B-cell proliferation and malignant transformation (102, 103). Such chronic antigen-mimetic stimulation drives continuous B-cell proliferation, substantially increasing the probability of DNA replication errors and mutational events during cell division. Concurrently, autoantibody-induced expression of activation-induced cytidine deaminase promotes genomic instability during somatic hypermutation and class-switch recombination, ultimately driving malignant transformation (104, 105).

Immune complex-mediated mechanisms. Autoantibodies can also exert oncogenic effects through immune complex formation. Circulating autoantibodies combine with their respective antigens to form immune complexes that deposit in target tissues such as lymph nodes, salivary glands, or synovium. These complexes activate the classical complement pathway and interact with Fc receptors, eliciting robust local inflammatory responses (106–108). This process recruits abundant neutrophils and macrophages, which in turn release reactive oxygen species (ROS), reactive nitrogen species (RNS), and pro-inflammatory cytokines (e.g., IL-1β, IL-6, TNF-α). The resulting oxidative stress and DNA damage, together with sustained proliferative and survival signals, create a permissive microenvironment for malignant transformation (109–111). In RA, anti-citrullinated protein antibodies (ACPAs/anti-CCP) form immune complexes with citrullinated antigens, a process thought to drive both local and systemic inflammation while contributing to lymphomagenesis (112, 113).

#### 4.2.2 Key pathways revealed by gene expression profiling

The application of gene expression profiling (GEP) has provided a new systems biology perspective on the molecular mechanisms of ADs-associated lymphoma. Large-scale transcriptomic analyses based on public repositories such as GEO have demonstrated numerous differentially expressed genes (DEGs) in AD-related lymphoma patients. These DEGs are significantly enriched in key biological processes, including immune regulation, cell cycle progression, programmed cell death, and DNA damage responses, thereby highlighting the distinct molecular characteristics of lymphomas arising in autoimmune contexts (114–116).

Of particular note, integrative analyses have consistently identified aberrant activation of the IL-17, TNF, and oestrogen signalling pathways in ADs-associated lymphoma patients, suggesting that these pathways play pivotal roles in disease pathogenesis. IL-17 pathway activation promotes differentiation and recruitment of Th17 cells, thereby sustaining chronic inflammation and perpetuating pro-inflammatory cytokine networks (including IL-6, IL-8, GM-CSF), which create a tumour-supportive microenvironment (117, 118). The TNF pathway, via NF-κB and JNK cascades, regulates cell survival, proliferation, differentiation, and apoptosis. Its persistent activation is not only a hallmark of chronic inflammation but also a major driver of genomic instability and malignant transformation (119, 120). In addition, dysregulation of the oestrogen signalling pathway may explain the striking sex bias observed in certain ADs (e.g., SLE, SjD, both predominantly affecting females). By modulating B-cell development, antibody production, and inflammatory responses, oestrogen signalling contributes to lymphoma risk under autoimmune conditions (121, 122).

Collectively, these findings extend our systemic understanding of the oncogenic role of autoantibodies and associated signalling pathways in ADs-related lymphomas. They also provide a theoretical foundation for risk stratification models and the development of novel targeted therapies.

### 4.3 Systematic assessment of risk factors

The development of ADs-associated lymphomas is shaped by a multidimensional risk network comprising intrinsic disease factors, treatment-related exposures, and interactions between environmental and genetic determinants. Intrinsic disease characteristics provide the baseline risk, including disease activity and duration, specific autoantibody profiles, and patterns of organ involvement, which collectively reflect the cumulative effects of immune dysregulation. Treatment-related risk factors highlight the complexity of clinical management, as conventional immunosuppressants, glucocorticoids, and biologics, while controlling disease activity, may also influence lymphomagenesis through mechanisms such as genotoxicity or pathway activation, creating a “therapeutic dilemma.” Environmental and genetic factors complete the risk landscape, with contributions from smoking, chemical exposure, infections, and genetic susceptibility. These three layers of risk factors do not act independently but interact dynamically to form a network that collectively drives lymphoma development in the context of autoimmunity, underscoring the need for holistic and individualised risk assessment and management strategies in clinical practice.

#### 4.3.1 Intrinsic disease factors: the cumulative effect of immune dysregulation

Intrinsic disease factors constitute the fundamental risk elements for ADs-associated lymphoma development. The core mechanism lies in the cumulative effects of persistent abnormal immune system activation. The distinction from the previously discussed mechanisms centres on the “pattern of risk accumulation” and “clinically quantifiable indicators” (123–126).

Disease activity and duration are pivotal dimensions of cumulative risk. In RA, high inflammatory burden—manifested by markedly elevated C-reactive protein (CRP) and rapid joint destruction—correlates with lymphoma risk (127). Patients with active and persistent RA inflammation may face up to a ninefold higher risk of NHL (127), and those with severe, long-standing RA may have as much as a 70-fold increase in risk (128). In SjD, characteristic lymphocytic infiltration of exocrine glands and polyclonal B-cell activation are observed (129). Approximately 30% of patients show extra-glandular manifestations (e.g., salivary gland swelling, lymphadenopathy, splenomegaly), which are associated with more severe, active disease and an elevated risk of lymphoma (58, 130–133). The median interval between SjD diagnosis and lymphoma development is about one to eight years, a relatively short window that provides a critical opportunity for malignant transformation (134).

Autoantibody profiles are important markers for tumour risk stratification. Known high-risk antibodies include anti-TIF1-γ antibodies commonly seen in dermatomyositis (135, 136) and anti-RNA polymerase III antibodies strongly associated with systemic sclerosis (98, 137, 138). Interestingly, in systemic sclerosis, patients negative for anti-centromere, anti-topoisomerase I, and anti-RNA polymerase III antibodies were reported to have higher cancer risk than antibody-positive counterparts, suggesting a complex balance in autoantibody-mediated risk modulation (139).

In summary, the high activity, prolonged disease course, and specific autoantibody profile of ADs collectively establish a risk network for lymphoma development through cumulative immune dysregulation effects. Clinically, monitoring these core indicators is essential for early identification and intervention in high-risk patients.

#### 4.3.2 Treatment-related risk factors: the double-edged sword of immunosuppression

The interplay between immunosuppressive therapy and lymphoma development in ADs exemplifies a “double-edged sword” (40). While immune dysregulation inherent to diseases such as RA and SLE is a major driver of lymphoma, chronic antigenic stimulation and inflammation confound attempts to disentangle disease-related risks from treatment-related effects (33, 140–142).

From a drug category perspective, tumour-related risk profiles exhibit significant heterogeneity among different immunosuppressants, with methotrexate (MTX) being the most systematically studied. MTX may induce MTX-associated lymphoproliferative disorders (MTX-LPD) through three mechanisms: First, it inhibits folate metabolism, disrupting purine and pyrimidine synthesis, thereby directly inducing DNA damage (143); Second, it downregulates T-cell immune surveillance, weakening the body’s ability to clear abnormal lymphocytes (144, 145). Third, it promotes Epstein-Barr virus (EBV) reactivation, driving abnormal lymphocyte proliferation (146). A study of RA patients receiving MTX treatment showed an MTX-LPD incidence rate of 2.64% (147), with risk closely correlated to higher drug doses (148). Pathological subtypes predominantly included DLBCL (40%-50%) and classical Hodgkin lymphoma (10%-30%) (149). In some cases, disease reversal occurred after MTX discontinuation, though malignant transformation risks persisted. Prognosis varied significantly by histological subtype: MTX-LPD patients with DLBCL achieved an 81% remission rate post-discontinuation, whereas 76% of CHL patients failed to achieve disease control after stopping MTX, necessitating further chemotherapy intervention (150). The immunosuppressive effects of azathioprine may weaken the body’s immune control over EB virus, thereby increasing the risk of EBV-associated lymphoma (151, 152). Biologics, such as TNFi, remain controversial. Large prospective studies have not consistently shown a significant increase in overall lymphoma risk (141, 153). Proposed mechanisms include inhibition of activation-induced cell death (AICD), potentially fostering clonal expansion of T cells or γδT cells, particularly with long-term exposure (154–156). Glucocorticoids also exhibit dual effects: early use may lower risk by suppressing inflammation (157), whereas prolonged high-dose therapy may promote tumour progression (158).

Current consensus suggests that early and aggressive inflammation control is key to reducing lymphoma risk (128, 159). Yet, prolonged use of certain immunosuppressants can induce monoclonal lymphocyte proliferation. A classic example is MTX-LPD, characterised by clonal expansion of EBV-infected B cells. While some cases regress after drug withdrawal, others may progress to lymphoma. This drug-induced monoclonal proliferative potential may partially offset the long-term benefits gained from controlling inflammation (160, 161). Therefore, clinical decision-making must balance the immediate threat of uncontrolled inflammation against the long-term carcinogenic potential of therapy, tailoring treatment strategies to optimise the risk–benefit profile across the disease course (162).

#### 4.3.3 Environmental and genetic interactions: a multifactorial oncogenic network

Environmental exposures and genetic predisposition synergistically shape the multifactorial oncogenic network underlying ADs-associated lymphomas. Environmental drivers substantially elevate lymphoma risk (163). Smoking, a recognised risk factor for both RA and lymphoma (56, 164), contributes through chronic inflammation, immune dysregulation, and microenvironmental alterations that collectively facilitate lymphomagenesis (165). Occupational exposures, including long-term contact with benzene-based solvents and pesticides, contribute to malignant lymphocyte transformation via direct DNA damage and epigenetic modifications (166–169).

Pathogenic infections act as critical co-factors in the context of immune dysregulation. ADs patients, prone to persistent infections due to immune dysfunction, are more vulnerable to oncogenic viruses such as EBV and human papillomavirus (HPV), perpetuating a vicious cycle of “immune deficiency–chronic infection–lymphoma transformation” (170–173).

Genetic susceptibility provides the baseline layer of individual risk. Family studies indicate that individuals with first-degree relatives affected by lymphoma face a 1–3-fold elevated risk (174, 175). Specific HLA alleles, through their roles in antigen presentation and immune recognition, are subtype-specific genetic risk factors for lymphoma (176). Genome-wide association studies (GWAS) further highlight shared susceptibility loci between ADs and lymphoma, particularly in regions regulating immune modulators such as TNF-α and IL-10. These variants influence the magnitude and duration of immune responses, thereby modulating phenotypic risk (177–179). Collectively, environmental exposures, infections, and genetic predispositions interact through a highly complex network that drives lymphoma progression in the autoimmune setting.

## 5 Treatment and prognosis of autoimmune disease-associated lymphomas

The treatment of ADs-associated lymphomas is complex and necessitates multidisciplinary collaboration. Currently, no unified standard therapeutic protocol has been established, and clinical decision-making largely depends on the histological subtype, clinical stage, aggressiveness of the lymphoma, and the activity of the underlying autoimmune disease (38, 40). Therefore, treatment must first adhere to the principle of individualised therapy for specific lymphoma subtypes (such as DLBCL, MALT lymphoma, HL, etc.). Traditional first-line regimens, such as CHOP (cyclophosphamide, doxorubicin, vincristine, prednisone) or CVP (cyclophosphamide, vincristine, prednisone) regimens, with or without rituximab and radiotherapy, remain the cornerstone of treatment for many aggressive B-cell lymphomas (6, 180). For indolent MALT lymphoma, particularly in patients with SjD, local radiotherapy, rituximab monotherapy, or even close observation in certain strictly selected low-burden cases are all reasonable options (43, 181, 182).

However, given the inherent immune dysfunction in these patients, treatment strategies exhibit distinct characteristics compared to primary lymphomas, particularly regarding treatment safety. Patients with AD-associated lymphoma face heightened risks of treatment-related toxicity when undergoing cytotoxic chemotherapy, constituting a core challenge in clinical management (8, 183). This increased risk stems from multiple overlapping factors: persistent underlying immune inflammation may compromise organ functional reserve (e.g., renal function in SLE patients) (184); and weakened immune system function due to long-term use of glucocorticoids and other immunosuppressive agents (185, 186). Furthermore, ADs-associated lymphoma patients may encounter unique challenges during chemotherapy, as their underlying immune dysfunction and prior immunosuppressive therapy may increase infection risk. Studies indicate that ADs patients receiving R-CHOP therapy may experience higher rates of febrile neutropenia compared to standard lymphoma patients, necessitating more aggressive infection prevention strategies (4). For lymphoproliferative disorders induced by specific immunosuppressive agents like methotrexate, clinical practice prioritises reducing or discontinuing these drugs as the primary management approach, leading to disease remission in some patients (187).

Current research and clinical practice are increasingly favouring novel targeted drugs and cell therapies that target the B-cell pathway (188–191). Among targeted therapies, anti-CD20 monoclonal antibodies (rituximab) play a pivotal role. Rituximab not only directly eliminates CD20-positive B-cell lymphoma cells but also indirectly controls autoimmune disease activity by depleting the aberrant B-cell pool (192–194). Clinical evidence suggests that the R-CHOP regimen in patients with ADs-associated NHL achieves high complete remission (CR) rates while simultaneously improving clinical manifestations and immunological parameters of coexisting conditions such as RA (180). The advent of Bruton’s tyrosine kinase (BTK) inhibitors (e.g., ibrutinib, zanubrutinib) provides new therapeutic opportunities (188). These agents block BTK, a critical mediator of BCR signalling, which plays an essential role in the pathogenesis of both B-cell lymphomas and autoimmune diseases (195). For high-risk, relapsed, or refractory cases, HSCT, particularly autologous HSCT (AHSCT), serves as an intensive treatment option (61, 196). Case reports describe long-term remission of concurrent autoimmune disorders, such as Crohn’s disease, following AHSCT or even allogeneic HSCT, strongly suggesting shared pathogenic mechanisms between the two conditions (197). Nevertheless, instances where lymphoma is cured but the autoimmune disease persists necessitating ongoing pharmacotherapy have also been reported (198, 199). Therefore, indications for transplantation must be carefully defined, and it is generally prioritised for controlling aggressive lymphomas.

The prognosis of ADs-associated lymphomas remains controversial, reflecting its inherent complexity. Several studies have identified a history of ADs, particularly B-cell-mediated diseases such as RA, SLE, and SjD, as an independent adverse prognostic factor for B-cell NHL, associated with shorter relapse intervals, reduced relapse-free survival (RFS), and overall survival (OS) (8, 24). The underlying mechanisms may include sustained immune activation, a pro-tumour inflammatory microenvironment, and impaired immune surveillance. Conversely, a prospective cohort analysing eight autoimmune diseases associated with lymphoma reported no overall impact of ADs on event-free survival (EFS) or OS. Interestingly, in B-cell-mediated ADs such as RA, autoimmune status correlated positively with EFS in marginal zone lymphoma and Hodgkin lymphoma, whereas no such association was observed in T-cell-mediated ADs (2). In contrast, other studies have failed to demonstrate a significant adverse prognostic impact of ADs themselves (200). These discrepancies highlight the importance of accounting for potential confounding effects of prior treatments, particularly high-dose corticosteroids or immunosuppressants. Moreover, the widespread use of agents such as rituximab, which possess both anti-tumour and immunomodulatory properties, has markedly improved outcomes in subsets of patients with ADs-associated lymphomas, potentially contributing to temporal inconsistencies across studies.

In summary, the treatment of autoimmune-associated lymphomas requires a dual-pronged strategy. This involves both delivering standardised, precision antitumour therapy for specific lymphoma subtypes based on evidence-based medical guidelines, and meticulously managing the underlying immune disorder throughout the treatment course. Additionally, heightened vigilance is essential regarding the elevated treatment-related risks stemming from the immunological background and prior treatment history. Prognosis assessment remains challenging, influenced by disease subtype, treatment modality, and evolving medical paradigms. Future research urgently requires rigorously designed prospective clinical studies to elucidate the biological essence of these diseases and explore more targeted, personalised treatment strategies, ultimately improving long-term quality of life for this unique patient population.

## 6 Conclusions and perspectives

Research into ADs-associated lymphomas has emerged as a critical interdisciplinary field bridging rheumatology and oncology. The relationship between ADs and lymphoma underscores the dual role of the immune system in oncogenesis. On one hand, the pro-inflammatory state inherent to ADs—characterised by chronic antigenic stimulation, cytokine release, and genomic instability—directly fosters malignant transformation. On the other hand, immunosuppressive therapies, while essential for controlling autoimmune inflammation, may inadvertently increase lymphoma risk by impairing immune surveillance, reactivating oncogenic viruses (e.g., EBV), or inducing clonal proliferation. This seemingly counterintuitive interplay highlights the therapeutic dilemma in clinical management: achieving a delicate balance between effectively suppressing the underlying autoimmune activity and minimising treatment-related oncogenic risks.

This review synthesises existing evidence from epidemiology, molecular mechanisms, and clinical management, underscoring that the pathogenesis of ADs-associated lymphomas is a multistep, multifactorial process. It is driven by chronic inflammation, underpinned by genetic susceptibility, triggered by environmental factors, and modified by therapeutic exposures. The central mechanism involves persistent immune activation disrupting the delicate balance between autoimmunity and malignant transformation, with TLSs and specific autoantibodies acting as key intermediaries.

Despite significant advances in recent years, numerous challenges remain. Future research should prioritise the development of risk prediction models informed by multi-omics data, integrating genomics, epigenomics, transcriptomics, and microbiome analyses to enable early identification and precision intervention in high-risk individuals. Detailed characterisation of the dynamic cellular interactions within TLSs, aided by single-cell and spatial transcriptomic technologies, is crucial for elucidating the molecular and cellular events driving malignant transformation. Exploration of innovative treatment strategies is equally important, including evaluation of bispecific antibodies, *in vivo* CAR-T cell therapies, and mitochondria-targeted agents, with a focus on restoring immune balance rather than solely suppressing or activating immunity (96). Furthermore, future research should aim to identify optimal therapeutic windows and personalised strategies that maximise lymphoma risk reduction. Establishing large prospective cohorts and real-world study platforms for long-term monitoring of patients’ immune repertoires and clonal evolution trajectories will facilitate the discovery of predictive biomarkers.

Ultimately, optimisation of multidisciplinary care pathways will enable comprehensive, individualised management strategies for ADs patients—from early risk surveillance and preventive interventions to lymphoma treatment and long-term follow-up. Such an approach not only holds the promise of improving outcomes for this special patient population but also offers a new paradigm for cancer immunoprevention, paving the way toward transformative clinical practice.
